# Adaptive Reflection on Negative Emotional Experiences: Convergences and Divergence Between the Processing-Mode Theory and the Theory of Self-Distancing Reflection

**DOI:** 10.3389/fpsyg.2019.01943

**Published:** 2019-09-03

**Authors:** Félix Cova, Felipe Garcia, Cristian Oyanadel, Loreto Villagran, Dario Páez, Carolina Inostroza

**Affiliations:** ^1^Departamento de Psicología, Universidad de Concepción, Concepción, Chile; ^2^Facultad de Ciencias Sociales y Comunicación, Universidad Santo Tomas, Concepción, Chile; ^3^Facultad de Psicología, Universidad del País Vasco, Leioa, Spain; ^4^Facultad de Educación y Ciencias Sociales, Universidad Andres Bello, Concepción, Chile; ^5^Departamento de Psiquiatría y Salud Mental, Universidad de Concepción, Concepción, Chile

**Keywords:** reflection, rumination, negative emotional experiences, processing-mode theory, theory of self-distancing reflection

## Abstract

Reflecting on negative emotional experiences can be adaptive but it can also maintain or intensify detrimental emotional states. Which factors determine whether reflection can have one consequence or another is unclear. This study focused on two research programs that have concentrated on this topic in the last decades: processing-mode theory (PMT) and self-distancing theory (SDT). The article described and contrasted both programs and their findings. The promising results that PMT and SDT have achieved in identifying the differences between the forms of adaptive and maladaptive reflection are highlighted. Likewise, the disconcerting contradictions observed between both programs that make integrating the findings difficult are indicated. The PMT states that adaptive reflection is concrete, and it is focused on the how of the experience. The SDT states that adaptive reflection is self-distanced and focused on the global meaning of the experience. The article finishes by indicating possible explanations for these apparent contradictions and outlines the challenges to be solved to improve comprehension of the topic.

## Introduction

Reflecting on experiences that imply emotional discomfort, sadness, or psychological pain (hereinafter “negative emotional experiences”) is a form of usual self-reflection and, to some degree, almost unavoidable. Notably, reflecting is usually necessary for properly processing vital experiences (Stanton and Low, 2012). By contrast, attempting to suppress or avoid thinking about negative emotional experiences is frequently self-defeating (Webb et al., 2012).

Many psychotherapeutic practices with proven effectiveness are supported and favor reflection on negative emotional experiences (Greenberg, 2017). Writing about negative emotional experiences is a process that implies reflection and has been shown to be positively related to better adaptation (Frattaroli, 2006; Hoyt et al., 2016). Some forms of reflection (e.g., “deliberate rumination”) have been shown to favor learning and “post-traumatic growth” processes after adverse experiences (Garcia et al., 2016). However, diverse research has shown that reflection on negative emotional experiences can also have detrimental effects and maintain or intensify negative emotional states (Mor and Winquist, 2002; Nolen-Hoeksema et al., 2008). This apparent paradox has resulted in attempts to differentiate forms of self-focus and adaptive and maladaptive self-reflection with no clarity until now (Trapnell and Campbell, 1999; Siegle et al., 2004; Watkins, 2008; Kross and Ayduk, 2017; Nakajima et al., 2018).

The reflection on negative emotional experiences can be understood as one of many forms of repetitive thinking. Repetitive thinking corresponds to a broad and encompassing term proposed by Watkins (2008) to refer to different process forms of prolonged or recurrent thought about one’s self, concerns, and experiences. A wide variety of terms are available for forms of repetitive thought, and it is not always clear whether these terms refer to different processes or are different names of analogous processes (Webb et al., 2012). Examples of these terms include rumination (with various acceptations), cognitive and emotional processing, perspective-taking, simulation and mental imagery, preoccupation, problem-solving, and reassessment. These terms have different degrees of conceptual overlap with self-reflection.

The study of the reflection on negative emotional experiences, and expressly using this term, has been mainly developed in the context of response styles theory (Nolen-Hoeksema et al., 2008). Although this theory focuses on rumination, regarding development, it distinguishes two rumination components: a reflection component (pondering reflection) and a more self-judging component (brooding) (Treynor et al., 2003). In this perspective, rumination is understood as “a mode of responding to distress that involves repetitively and passively focusing on symptoms of distress and on the possible causes and consequences of these symptoms” (Nolen-Hoeksema et al., 2008, p. 400). This form of rumination has been called “depressive rumination” (Papageorgiou and Wells, 2004), and it is generally understood as focusing on the thought of the emotional discomfort experience and the events and situations related to those discomfort states (Watkins, 2004, p. 6).

Depressive rumination has been shown to be a predictor of different negative consequences for individuals, increasing emotional discomfort, and appears as a risk factor for diverse mental disorders (Trick et al., 2016). The pondering reflection component was defined as “a purposeful turning inward to engage in cognitive problem solving to alleviate one’s depressive symptoms” (Treynor et al., 2003, p. 256), whereas brooding was defined as “a passive comparison of one’s current situation with some unachieved standard” (Treynor et al., 2003, p. 256), implying more self-deprecating and regretting components. Diverse research has shown that the clearly negative component of rumination is brooding (Joormann et al., 2006; Chan et al., 2009; Cova et al., 2009; Cox et al., 2012; Romero et al., 2014; Xavier et al., 2016).

Although the distinction between reflecting and brooding is useful to suggest clues about the aspects that would differentiate more adaptive and maladaptive thought processes of negative emotional experiences, the literature has shown that is insufficient for a broader clarification of these differences. Notably, pondering reflection does not show relations with positive adaptation processes like post-traumatic growth (García et al., 2017), and in some studies, it has been shown to be a maladaptive process, but not at the brooding level (Miranda and Nolen-Hoeksema, 2007; Gooding et al., 2012; Hasegawa et al., 2013). The research into the topic has been limited by the scarce conceptual elaboration regarding what is pondering reflection (and by the limitations of the scale used to measure it) (Rude et al., 2007). Thus, the developments of the response styles theory allow it to show that in a case where the rumination takes the form of brooding, it is clearly maladaptive, but they do not allow for the identification of whether there is another form of thinking of negative emotional experiences other than brooding that can be necessary or beneficial. Thus, although the term rumination is used to refer to a maladaptive form of thinking on negative emotional experiences, it is rather brooding and their analogous processes the ones that have that profile. Reflection and rumination may be considered as broad terms and synonyms to refer to the focus of thinking on negative events and emotional experiences, where processes that can be adaptive or maladaptive coexist.

The study on the types of reflection about negative emotional experiences can be placed in a broader context of research on coping strategies for adverse events, emotional regulation, and cognitive and emotional processing. One of the most relevant challenges has been the attempt to differentiate those strategies and processes that seem, in general, more adaptive and less adaptive (Páez and Costa, 2014). The meta-analysis presented by Webb et al. (2012) identified reappraisal as the emotional regulation strategy with the highest positive effect (specifically, reappraisal in the form of perspective-taking, although, reappraisal of the answer – analogous to acceptance – and reappraisal of the emotional stimuli also showed positive effects). Likewise, diversion from and suppression of the emotional expression showed positive effects. Whereas, focusing on the emotion (e.g., “let yourself feel the event as if you were there”) as well as on the causes and implications of the events (e.g., “think about why you react the way you do”) showed negative effects. Another meta-analysis of the relation between emotion-regulation strategies and psychopathology showed that reappraisal and acceptance have an adaptive role; in contrast, rumination, suppression, and avoidance have a maladaptive one (Aldao et al., 2010). These meta-analyses have not considered strategies such as problem-solving and mindfulness, which have also shown to be useful in reducing the impact of negative events (Schäfer et al., 2017; Iani et al., 2019).

These generalizations of the adaptive or maladaptive value of coping and emotion-regulation strategies must be taken with caution since this judgment depends on various factors, such as individual differences and the moment and context in which strategies are used (Naragon-Gainey et al., 2017). However, these generalizations allow an approximation to comprehend which strategies are, in general, more useful for people when facing negative events and emotions.

The purpose of this article is to conduct a conceptual analysis of two intensive and extensive research programs which focus specifically on reflection on adaptive and maladaptive processes. One program is based on the **processing-mode theory, PMT** (the main author is E. Watkins), and the other on the theorization of the self-distanced analysis, self-distancing theory, **SDT** (the main authors are Ayduk and Kross). These are broad research programs that have provided solid empirical evidence to support their proposals (Watkins, 2008, 2016; Kross and Ayduk, 2017; Ayduk and Kross, 2018, for thorough reviews). A review of the publications related to PMT and SDT was carried out. The reviews made by the authors of both theories were used as the initial source for the current study. Moreover, literature searches in the electronic data bases of PsycINFO, Web of Science, and Scopus were carried out using “concrete rumination,” “abstract rumination,” “processing mode,” “self-distance,” and “self-immersion” as keywords (from 2005 to 2019). Articles and books of interest cited in the reviewed publications were also considered.

In this paper, we mainly focused on the most important findings of each program and the description and analysis of their similarities and differences. Few publications were found regarding the relations between both lines of research (Kross and Ayduk, 2008; Rude et al., 2011). We considered this surprising due to the importance of both programs and their mutual implications.

## E. Watkins’ Program: Processing-Mode Theory

Watkins (2004, 2008, 2013, 2016) made a great effort to distinguish adaptive repetitive thought forms from maladaptive repetitive thought forms. He proposed three factors that would be crucial in the distinction between adaptive and maladaptive forms of repetitive thought: (1) the content of the thought, (2) factors of the intrapersonal and interpersonal context where this occurs, and (3) the processing mode (Watkins, 2008). The forms of repetitive thought whose contents are negative aspects of the experiences and about oneself are subject to an increased risk of being detrimental, although that does not exclude that certain forms of thought of negative emotional experiences could be adaptive. That would depend on the intrapersonal and interpersonal context where they are performed and the processing mode.

Intrapersonal and interpersonal contexts may affect thought processes. The influence of the intrapersonal context is illustrated by the difference between thinking of negative emotional experiences and being a person who has high or low self-esteem. The same occurs in the interpersonal sphere, for example, an analysis of an experience in a vital situation of high or low stress will differ.

Concerning the PMT, it distinguishes abstract and concrete processing modes using the construal level theory (Trope and Liberman, 2003). “An abstract processing mode is conceptualized as focusing on general, superordinate, and decontextualized mental representations that convey the essential meaning, causes, and implications of goals and events (…)” (Watkins, 2016, p. 388). Although this abstract mode provides stability to goals and representations, the abstract mode makes an individual less responsive to the context and provides merely a few specific guidelines for the action; then, the opposite occurs in a concrete level of construct. Although these two thought-processing modes are necessary for individuals, the use of one or the other to reflect on negative emotional experiences would have different implications.

### Abstract Reflection or Rumination

This form of repetitive thought implies an analytical and evaluative process focused on the comprehension of the causes of an event and its consequences. In a case when this form of repetitive thought is used to analyze negative emotional experiences, it would be maladaptive. Schematically, this form of repetitive thought focuses on questions related to the why (e.g., “Why did it happen?”), that is, a question that would direct attention toward the abstract evaluation of the experiences and situations lived and favor the probability that the individual adopts a global and negative explanatory style and persistently thinks about her/his problems in this manner (Bassanini et al., 2014).

### Concrete Reflection or Rumination

This form of repetitive thought implies a processing mode focused on specific and contextual detail, direct experience in the present moment, and the process of how things happen. The thought is organized around questions based on the “how” of the experience lived, and not the “why” (Watkins et al., 2008). By being more concrete and focused in the process, it would be also more directed to the search for solutions (Watkins and Moulds, 2005). It would be a form of adaptive reflection. “Focus on the direct, specific, and contextualized experience of an event, and on the details of goals, events, and actions that denote the feasibility, mechanics, and means of how to do the action” (Watkins, 2016, p. 30). “This functional counterpart is often focused on finding an active, concrete and immediate solution” (e.g., “How could I cope with this problem?”) (Bassanini et al., 2014, p. 19).

Watkins’ conceptualization of concrete rumination implicitly integrated two different components: one more experiential and the other more cognitive problem-solving oriented. Sometimes, he characterizes it by its experiential aspect, for example, when he describes that it implies an individual should “focus attention on the experience of feelings, mood, and symptoms” (Watkins et al., 2008). One statement that induces concrete rumination is as follows: “I would like you to focus on how it happened, and to imagine in your mind as vividly and concretely as possible a ‘movie’ of how this event unfolded” (Watkins et al., 2008, p. 366). At other time, the author characterizes it more as a process where a situation is analyzed in detail, including how to manage it (Watkins, 2016). This second aspect is considered in one of the standardized procedures developed to assess concrete rumination, for example. In this procedure, individuals must state which relation they identify, between their own thought modes, when facing a negative event and the one shown by a comic-strip character. In the comic strips in which the character concretely processes, her/his thoughts are “How unlucky I am! How can I handle this, now? Which is the best solutions I can get? What if G was in this situation? The best thing I can do is minimize the delay, which alternative strategies can I use?” (Bassanini et al., 2014, p. 20). This comic strip shows a way of understanding the concrete rumination very closed to what it would be problem-focused coping and with no “experiential” component.

There are two complementary lines of evidence for the PMT. The first is the experimental research about the effects of inducing the two forms of thought. Instructing the individuals to engage in concrete rumination produced a faster recovery from negative effects and reduced intrusions after a previous negative induction (Watkins, 2004; Watkins et al., 2008; Ehring et al., 2009). Stimulating concrete rumination in individuals with a diagnosis of depressive disorder reduces their negative global judgment (Rimes and Watkins, 2005) and increases their skills for problem-solving (Watkins and Moulds, 2005) and the specificity of their recalls (Watkins and Teasdale, 2001). By contrast, the analysis of the causes and meanings of their experience increases overgeneralization, avoids problem-solving, and increases depressive mood (Watkins, 2016).

The second line of evidence has been the development of depression treatment and prevention programs focused on the modification of the abstract processing, and an increase in the concrete processing mode. In the clinical trials performed, the training in “concrete rumination” has been shown to be effective in reducing abstract rumination such as depressive and anxious symptoms and disorders (Watkins and Moulds, 2007; Watkins et al., 2009, 2011, 2012; Topper et al., 2017).

## O. Ayduk and E. Kross’ Program: Self-Distancing Theory

This line of research was originally based on the distinction between cold and hot processing forms and the concept of “psychological distance” (Metcalfe and Mischel, 1999; Kross et al., 2005). In this proposal, hot representations elicit reflective processing that is predominantly under stimulus control, leading to automatic approach and avoidance behaviors, whereas, cool representations, cognitively driven, reflect processing that is more effortful and is instrumental in inhibiting automatic responses activated by hot representations. From these initial conceptualizations, the authors proposed the existence of two differentiating dimensions of the reflection about negative emotional experiences: (1) the type of perspective about the self from which the experience is analyzed and (2) its content. Regarding the type of perspective, they made a distinction between a self-immersed analysis and a self-distanced analysis. By self-distancing, authors understand a means to get close to their experience as an external observer. There are different modes in which this distancing can be experienced, for example, visually (seeing yourself from the outside) and how to refer to the experience or oneself (in third person).

More recently, authors have been exploring how the temporal perspective from which the experience is “observed” (e.g., from the future) can also imply distancing (Bruehlman-Senecal and Ayduk, 2015; Kross and Ayduk, 2017; Ranney et al., 2017), whereas the self-immersed analysis implies that the individual experiences the emotions and events again as they happened from a first-person perspective. Regarding the content of the thoughts, they distinguish an orientation toward the emotional experience (focus on the “how”) or toward the analysis of the experience (focus on the “why”). In the analysis conducted by Ayduk and Kross, the type of perspective and the content are related because asking yourself the why of the experience would ease a distanced perspective, and the attention to the how would ease self-immersion. From this model, the adaptive reflection is self-distanced and focused on the why. In that sense, a deciding factor is not whether the attention is put on the “why” or to the “how” but whether the reflection is self-distancing. We must emphasize that the attention to the why, however, is not accessory: the search for explanations for the negative experiences is, from the authors’ point of view, an epistemic need of human beings; therefore, performing it in an adaptive way is crucial (Ayduk and Kross, 2018).

Another aspect we want to highlight is the distinction the authors made between the self-distancing of negative emotional experiences pure and simple, which can have various purposes and uses, and the self-distanced analysis, which implies directing the cognitive process to the analysis of the experiences (Kross et al., 2012). In some cases, pure and simple self-distancing can be negative, for example, when it is a chronic form of avoidance. The authors emphasize that the self-distanced analysis of the experience is what is adaptive and not the distancing *per se*. Self-distancing reflection would allow a modification of the experience, which stops being recalled time after time because its reconstruction has been allowed, easing the feeling of insight and closing (Kross and Ayduk, 2008).

Studies of natural patterns of the use of self-immersion and self-distancing, as well as experimental studies, have shown that self-distanced reflection is more adaptive: less negative emotional activation immediately and in follow-ups, less depressive rumination, and more positive physiological responses such as lower levels of blood pressure (Ayduk and Kross, 2008, 2010; Kross and Ayduk, 2008). Studies of individuals with depressive symptomatology and individuals with a diagnosis of major depressive disorder have shown similar outcomes (Kross and Ayduk, 2009; Kross et al., 2012). Recently, studies of the efficacy of the training in self-distancing reflection have been developed, with promissory results in the expected direction (Penner et al., 2016; Orvell et al., 2017). Indirect support to these authors’ approaches comes from the results of the meta-analysis presented by Webb et al. (2012), where reappraisal *via* perspective-taking appears as the form of emotion-regulation with the highest adaptive value. This view of reappraisal is very close to the self-distanced analysis (e.g., “increase your sense of objective distance, viewing pictured event from a detached third-person perspective”).

## Contrast Between the Research Programs

We observed that both research programs have the same interest in identifying adaptive reflection forms and differentiate them from what would be a non-adaptive form of reflection. In this context, both programs coincide in identifying brooding as a clearly maladaptive form of rumination. However, the reasons for that are somehow different: for PMT, the reason is the abstract nature, superordinate, of the focus of the thought in the negative emotional experience, which would be the maladaptive factor of brooding because it leads to despairing and self-critical generalizations of the events and blocks the search for solutions. For SDT, the maladaptive aspect of brooding is the absence of self-distancing when thinking of negative emotional experiences because that favors the “emotional overflow” and avoids the processing and reconstruction of the experience.

Differences between PMT and SDT are even clearer at the moment of analyzing how adaptive reflection is conceptualized. Terms used in each program are eloquent in this regard: PMT identifies adaptive reflection with the analysis of the “how” of the experience and not the “why,” unlike SDT. For SDT, a more abstract and comprehensive analysis of the experience, provided that it is self-distancing, is more adaptive (although Ayduk and Kross initially described adaptive reflection as abstract, they decided later to avoid this term because they concluded that there would be non-adaptive, self-immersed, and other adaptive, self-distancing abstract processing modes; Kross et al., 2012). For PMT, the abstract processing of the negative emotional experience is always maladaptive. For PMT, the risk of thinking from the “why” regarding the negative emotional experiences is that it would reduce the activity and the search for solutions (Watkins, 2016). However, for SDT, thinking from the why is a facilitator of the negative emotional experience reconstruction to the extent that it favors self-distancing but recognizes that there can be an analysis of the why from the self-immersed perspective, which would be maladaptive and typical of brooding (Kross et al., 2012).

These differences between both programs, regarding the value of the abstraction and the analysis of “why” and “how” of negative emotional experiences, are reflected in how both use the construal level theory. SDT (Kross and Ayduk, 2017) considers that a high construal level, when analyzing negative emotional experiences, is positive because it favors psychological distance. PMT, by contrast, considers that a high construal level processing, when analyzing negative emotional experiences, is characteristic of maladaptive rumination (Watkins, 2008).

The authors of both research programs have not contrasted their proposals. Watkins (2008) has stated only that there are no analogies between the PMT and SDT concepts because PMT would refer to the differences in the construal levels of concrete and abstract rumination and SDT would refer to cold and hot processing, which would be at another analysis level. However, this observation made by Watkins is debatable: the relation between self-distanced analysis and cold processing, and self-immersed analysis and hot processing, was merely the origin of Ayduk and Kross’ conceptualization. Notably, these authors no longer use the terms “hot” and “cold” to characterize the two analysis modes they described. Self-distanced analysis is not cold because it implies the emotion, but this emotion is looked at or described from the “outside.” Kross and Ayduk (2008) have suggested that instructions used in Watkins’ studies to elicit concrete and abstract rumination favor self-distanced and self-immersed analysis, respectively.

Only one study related the concepts of both programs (Rude et al., 2011). This study stated that what PMT considers maladaptive rumination could not be characterized as an abstract rumination but as a negative assessment rumination. When comparing concrete rumination, evaluative abstract rumination, and non-evaluative abstract rumination (related to self-distancing reflection), the authors observed that the latter was the one that showed the most adaptive results.

## Discussion

Several researchers have shown a predominance of maladaptive effects of the reflection on negative emotional experiences (Mor and Winquist, 2002). In return, there is also strong evidence of the need and importance that reflecting on experienced negative emotions and events usually has (Greenberg and Pascual-Leone, 2006). This shows that the form and context of the reflective processes are determining factors of the type of effects that they produce. Negative emotional experiences stimulate the process of thoughts about them, and this probably occurs as part of self-regulation processes to search for solutions and processes to elaborate and integrate the experiences (Watkins, 2008; Vine et al., 2014). This phenomenon implies that negative emotional experiences can “activate” at the same time adaptive and maladaptive forms of reflection, which can be interconnected.

The lines of research developed by Watkins and by Ayduk and Kross have been focused in trying to distinguish the aspects of the focusing forms of thinking in the negative emotional experiences that would make them more adaptive or maladaptive. Both show positive results, but they characterize the implied processes in different ways. This sets a question of interest: how can concrete rumination and self-distanced analysis have similar positive effects if they correspond to forms of reflection understood differently and, in some respects, as the opposite?

At least three explanatory lines can be hypothesized. One possibility is that self-distancing reflection and concrete rumination avoid, through different ways, the tendency to use brooding and its detrimental effects. A second possibility is that both forms of processing allow an individual to block the negative effects of suppression and emotional avoidance and favor contact with maintained negative emotional states and the elaboration of their experiences. Notably, both programs obtained promising results in individuals with post-traumatic stress disorders, which supports this possibility (Sezibera et al., 2009; Wisco et al., 2015; Penner et al., 2016). A third possibility supposes that there would be more relation than it seems between concrete rumination and self-distanced analysis. Focusing on the “how” of the experience, as proposed by PMT, could result in a process of self-distanced analysis, depending on how this focus is operationalized. Notably, the treatment received by the individuals in “concrete rumination” in Watkins’ studies could be implying that individuals learn to analyze their experiences in a more distanced manner. Inversely, self-distanced analysis could be understood as a form of concrete rumination that allows the person to contact her/his emotional experiences, identifying specific aspects of the events that she/he is living and enabling new possibilities of action.

The study of these hypotheses requires differentiating the experiential component from the problem-solving component involved in “concrete rumination.” The experiential component is the most complex one since its effects seem to strongly depend on the way and context in which concrete rumination is made. In fact, from the SDT point of view, a detailed analysis of the emotional experience may favor self-immersion and it can have further detrimental consequences. The meta-analysis performed by Webb et al. (2012) supports the idea that an experiential focus on negative events and emotions is maladaptive – although less than focusing on the analysis of meanings and consequences of the experience. However, it can be hypothesized that, under certain circumstances, the experiential component may favor emotional approach and acceptance, and through this, having positive effects.

In this regard, notably, PMT and SDT state that there would be a relation between the processing mode they consider adaptive and mindfulness. Mindfulness is a form of attention to the distanced experience, where, unlike the explicit proposal of PMT and SDT, the experience is left to flow, with no analysis or judgment (Kross and Ayduk, 2017). Emotional distancing through mindfulness has been related to the decrease of emotional exhaustion (Karing and Beelmann, 2019). Mindfulness decreases the tendency to the abstract negative appraisal of the past and allows positive reappraisal (Desrosiers et al., 2013). In depressive patients treated with mindfulness-based cognitive therapy (MBCT), improvement is mediated by the increase in mindfulness and the decrease in maladaptive rumination (Shahar et al., 2010). Cluster analysis has shown the mindfulness core processes (describing and nonreactivity to the experience) are specifically related to acceptance and reappraisal as emotional regulation strategies, with positive outcomes (Iani et al., 2019).

Another possible relation between concrete rumination and self-distanced analysis is the easement of problem-solving. Problem-solving is explicitly stimulated in the training in concrete rumination. Self-distanced analysis also stimulates a broader view toward difficulties, which would allow to better identify the available solutions (Mori et al., 2015; Barth et al., 2016; Goldberg et al., 2019).

From our point of view, these three explicative lines are not incompatible. We think that self-distancing is at the basis of the effectiveness of concrete rumination. Also, self-distancing appears as a critical element to differentiate analysis forms focused in the causes and the meaning of the experiences that lead to brooding and intensify the discomfort of those that favor reappraisal and the integration of them in the narration and personal identity. However, we do not agree that self-distanced analysis must be always focused on the most global aspects and those aspects related to the meaning of the lived experiences. In certain contexts and moments, detailed approach to the experienced situations and emotional states can be convenient and necessary. On the other hand, the theory of the processing mode would be enriched if it considered that, at certain moments and circumstances, the analysis of the why and the meaning of negative emotional experiences could be a useful part of a reappraisal process of these.

Further researches are required to subject the stated hypotheses to test. A critical aspect is to determine how much self-distancing there is in concrete rumination and how much of concrete rumination there is in self-distanced analysis. It would be of particular interest to compare the effects of a self-distanced experiential analysis to the effects of a self-distanced analysis of causes and meanings in different situations and in different temporalities after an experience. Another related aspect of interest is that SDT leads to a belief that every implication in the emotional experience in the first person is negative emotional immersion, and does not necessarily have to be like that. Whether every emotional immersion is necessarily negative is unclear. That depends on how it is performed as shown, in the therapeutic field, by techniques such as prolonged exposure and other therapies focused on emotion (Greenberg and Pascual-Leone, 2006; Zandberg et al., 2017). Consequently, the identification of the temporal intrapersonal and interpersonal, and contextual factors that influence the reflection process is highly relevant.

In summary, PMT and SDT are research programs relevant to the identification of what is characteristic of the adaptive and maladaptive reflective processing of negative emotional experiences. A challenge is to better delimit the scope of their main approaches, to which the mutual contrast between them can contribute.

## Author Contributions

FC conceived of the presented idea and wrote the manuscript. FG, CO, DP and LV developed the conceptual and empirical background. CI was the main reviewer of the text. All authors discussed the results and contributed to the final manuscript.

### Conflict of Interest Statement

The authors declare that the research was conducted in the absence of any commercial or financial relationships that could be construed as a potential conflict of interest.
